# CRIPTO antagonist ALK4^L75A^-Fc inhibits breast cancer cell plasticity and adaptation to stress

**DOI:** 10.1186/s13058-020-01361-z

**Published:** 2020-11-13

**Authors:** Ozlen Balcioglu, Richard E. Heinz, David W. Freeman, Brooke L. Gates, Berhane M. Hagos, Evan Booker, Elnaz Mirzaei Mehrabad, Hyrum T. Diesen, Kishan Bhakta, Supraja Ranganathan, Masami Kachi, Mathias Leblanc, Peter C. Gray, Benjamin T. Spike

**Affiliations:** 1 Huntsman Cancer Institute, University of Utah, Salt Lake City, UT 84112 USA; 2 Department of Oncological Sciences, University of Utah, Salt Lake City, UT 84112 USA; 3 Peptide Biology Laboratory, Salk Institute for Biological Studies, La Jolla, CA 92037 USA; 4 Department of Biochemistry, University of Utah, Salt Lake City, UT 84112 USA; 5 Present Address: Biotheranostics Inc., San Diego, CA 92121 USA

**Keywords:** Breast cancer, Plasticity, CRIPTO, Stress adaptation, Cancer stem cells

## Abstract

**Background:**

CRIPTO is a multi-functional signaling protein that promotes stemness and oncogenesis. We previously developed a CRIPTO antagonist, ALK4^L75A^-Fc, and showed that it causes loss of the stem cell phenotype in normal mammary epithelia suggesting it may similarly inhibit CRIPTO-dependent plasticity in breast cancer cells.

**Methods:**

We focused on two triple negative breast cancer cell lines (MDA-MB-231 and MDA-MB-468) to measure the effects of ALK4^L75A^-Fc on cancer cell behavior under nutrient deprivation and endoplasmic reticulum stress. We characterized the proliferation and migration of these cells in vitro using time-lapse microscopy and characterized stress-dependent changes in the levels and distribution of CRIPTO signaling mediators and cancer stem cell markers. We also assessed the effects of ALK4^L75A^-Fc on proliferation, EMT, and stem cell markers in vivo as well as on tumor growth and metastasis using inducible lentiviral delivery or systemic administration of purified ALK4^L75A^-Fc, which represents a candidate therapeutic approach.

**Results:**

ALK4^L75A^-Fc inhibited adaptive responses of breast cancer cells under conditions of nutrient and ER stress and reduced their proliferation, migration, clonogenicity, and expression of EMT and cancer stem cell markers. ALK4^L75A^-Fc also inhibited proliferation of human breast cancer cells in stressed tumor microenvironments in xenografts and reduced both primary tumor size and metastatic burden.

**Conclusions:**

Cancer cell adaptation to stresses such as nutrient deprivation, hypoxia, and chemotherapy can critically contribute to dormancy, metastasis, therapy resistance, and recurrence. Identifying mechanisms that govern cellular adaptation, plasticity, and the emergence of stem-like cancer cells may be key to effective anticancer therapies. Results presented here indicate that targeting CRIPTO with ALK4^L75A^-Fc may have potential as such a therapy since it inhibits breast cancer cell adaptation to microenvironmental challenges and associated stem-like and EMT phenotypes.

## Background

The ability of tumor cells to reprogram their behavior in order to cope with environmental challenges is critical to tumor progression. Challenges include growth and survival in abnormal contexts during dissemination or at distant sites, or when normative signals are corrupted by tissue disruption, nutrient deprivation, or chemotherapy. Importantly, cellular and molecular mechanisms of plasticity that engender tumor cell persistence or even proliferation under microenvironmental-, therapeutic-, or metastasis-associated stress have begun to emerge as key therapeutic targets [1–4].

CRIPTO is a small GPI-anchored signaling protein that regulates normal stem cell activity and oncogenesis [5–8]. It is selectively upregulated in multiple human tumor types including ~ 80% of human breast cancers where it is associated with poor patient prognosis [9–11]. CRIPTO promotes anchorage-independent growth, migration, invasion, and EMT in mammary epithelial cells and breast cancer cell lines in vitro [11–13]. CRIPTO overexpression increases ductal branching, hyperplasia, and latent growth of tumors with an EMT-like phenotype in the mammary epithelium of mice [14–17]. In addition, CRIPTO knockdown inhibits proliferation of human MDA-MB-468 and SKBR3 breast cancer cell lines in vitro [18], while CRIPTO knockdown in a triple negative breast cancer (TNBC)-like transplantable mouse mammary tumor model blocks progression [19]. A CRIPTO mAb carrying a toxic payload has been shown to inhibit growth of MDA-MB-231 cells in a xenograft model [20]. CRIPTO has also been reported as a functional cancer stem cell marker in head and neck cancers and, more recently, in esophageal squamous cell carcinoma, hepatocellular carcinoma, and post-therapy, recurrent osteotropic prostate cancer [21, 22]. Several groups are seeking to develop CRIPTO as a biomarker of tumor aggressiveness and validate therapeutic modalities directed at blocking CRIPTO-induced oncogenesis [19, 23–25].

CRIPTO has multiple signaling functions. It is an essential co-receptor for certain TGF-β superfamily ligands including Nodal and attenuates signaling by others such as activin A, activin B, and TGF-β1 (reviewed in [5, 6]). CRIPTO also acts independently of its effects on TGF-β superfamily ligands as a soluble secreted factor that activates SRC and MAPK/PI3K pathways including activation of AKT [5, 6]. These signaling activities require CRIPTO binding to cell surface glucose regulated protein 78 kDa (GRP78), an endoplasmic reticulum chaperone that localizes to the cell surface under stress conditions and which, like CRIPTO, is overexpressed in breast cancers [26–32]. CRIPTO has also been reported to signal via WNT [33] and NOTCH [34] pathways providing additional mechanisms that may contribute to its roles in stem cells and oncogenesis.

We have developed a novel antagonist based on the CRIPTO binding properties of activin-like kinase 4 (ALK4), a transmembrane serine kinase receptor that mediates signaling by several TGF-β superfamily ligands including activins and Nodal [12]. This antagonist, ALK4^L75A^-Fc, is designed to have enhanced specificity for CRIPTO as it comprises a human Fc domain fused to a mutant ALK4 extracellular domain bearing a point mutation (L75A) that disrupts ALK4 binding to TGF-β superfamily ligands (i.e., activin and Nodal) without affecting its binding to CRIPTO [35]. We previously demonstrated that ALK4^L75A^-Fc specifically binds CRIPTO, inhibits CRIPTO signaling, and antagonizes CRIPTO-dependent maintenance of the normal mammary epithelial stem cell state [12]. In the present study, we demonstrate that ALK4^L75A^-Fc similarly inhibits stem cell phenotypes, plasticity, and adaptability of human breast cancer cells in vitro and in vivo, suggesting it may represent a viable therapeutic strategy for targeting facultative, tumorigenic, stem cell phenotypes that emerge as cancer cells respond to microenvironmental or therapy-associated stress.

## Methods

### Production of ALK4^L75A^-Fc

Purified ALK4^L75A^-Fc was produced as previously described [12] from 293T-conditioned media using sequential protein A and Flag affinity chromatography. For inducible ALK4^L75A^-Fc, the open reading frame reported in Spike et al. [12] was cloned into a custom 3rd generation lentiviral backbone (described in Supplemental Fig. 1). Lentiviral particles were packaged and concentrated according to the procedure described in Tiscornia et al. [36].

### Cell lines

293T, NCCIT, MDA-MB-231, and MDA-MB-468 cell lines were acquired from ATCC. Cells were cultured in “Tumor media” (Dulbecco’s modified Eagle’s media (DMEM) with ciprofloxacin 10 μg/ml, and 10% fetal calf serum), except NCCIT cells which were cultured in “NCCIT media” (RPMI 1640 medium, ATCC modification; GIBCO with ciprofloxacin 10 μg/ml, and 10% fetal calf serum). Cells were grown in sterile humidified tissue culture incubators at 37 °C with 5% CO_2_ and ambient (~ 17–18%) O_2_. Cells were transduced with lentiviral concentrates in the presence of 7 μg/ml polybrene for up to 16 h with transduction efficiency (not shown) suggesting MOI < 1. Cells were subsequently sorted for green fluorescence on a FACSAria fluorescence activated cell sorter (BD Biosciences, Franklin Lakes, NJ), following at least 3 days of culture. ALK4^L75A^-Fc expression was confirmed by western blot of cell lysates and immune-precipitation from conditioned media with protein A agarose beads (Abcam, Cambridge, UK, ab193254) and anti-Flag antibodies (Cell Signaling Technology, Danvers, MA, 8146). Inducible short hairpin vectors were acquired from Dharmacon, Inc. (Lafayette, CO, Cat# V3SH11255-01EG6997).

### Treatments

Low serum conditions were achieved by switching from each cell line’s standard media (above) to Opti-MEM (GIBCO, Waltham, MA) for 16 h. Cells were treated in vitro with purified ALK4^L75A^-Fc (10 μg/ml), recombinant mouse CRIPTO (0.3 μg/ml; R&D Systems, Minneapolis, MN), thapsigargin (Invitrogen, Carlsbad, CA) at 125 nM for MDA-MB-231 and 10 nM for MDA-MB-468 cells, 2-deoxy-d-glucose (4 mM; Sigma-Aldrich, St. Louis, MO), doxycycline (0.2–2 μg/ml; Sigma-Aldrich, St. Louis, MO), and LY290042 (10 μM; Sigma-Aldrich, St. Louis, MO) where indicated. A doxycycline dose of 1 μg/ml was used for in vitro cell treatments except where establishing doxycycline dose response (Supplement Fig. 1B).

### Flow cytometry

Single cell suspensions of cells were stained with antibodies to CD44 (AF647-anti-CD44, IM7; BioLegend, San Diego, CA) and surface GRP78 (AF488-anti-HSPA5, polyclonal rabbit; Novus Biologicals, Centennial, CO), and with DAPI. Viable (i.e., DAPI-negative) cells were gated for singlets, and the proportion of cells beyond a uniform threshold were enumerated under equivalent staining and voltages across samples. Aldefluor™ kit (StemCell Technologies, Vancouver, Canada) staining was conducted according to the manufacturer’s recommendation.

### Surface GRP78 detection

Surface GRP78 was detected in intact adherent cells by incubating cells on ice with primary GRP78-directed antibodies (N-20, Santa Cruz Biotechnology, Inc., Dallas, TX), followed by cold wash in PBS and incubation with 4% paraformaldehyde, followed by subsequent washing and mounting in the presence of DAPI. Cells were imaged on an SP8 white light confocal microscope (Leica Biosystems, Wetzlar, Germany).

### Cell proliferation and migration

To quantitatively measure proliferation, cells were equivalently seeded at low confluency and imaged every 2 h for the times indicated in an automated multi-well incubator/imaging apparatus Incucyte™ (Essen BioSciences, Ann Arbor, MI). Alternatively, cells were plated at density (50,000 cells per 96-well well), and scratch wounds were generated to allow longitudinal imaging in the Incucyte™. System intrinsic automated quantification of proliferation and wound filling was used. Cells were treated with Mitomycin C (Sigma-Aldrich, St. Louis, MO) at 40 μg/ml for MDA-MB-231 and 1 μg/ml for MDA-MB-468 cells.

### Methylcellulose cultures

Cells were suspended in 1% methylcellulose (R&D Systems, Minneapolis, MN) in tumor media and allowed to grow 10 days before analysis.

### Organoid serial passaging

MDA-MB-468 cells transfected with either the ALK^L75A^-Fc inducible lentiviral vector or a mock control were seeded in a 24-well low adhesion dish (Corning Inc., Corning, NY) at 5000 cells/well in tumor media supplemented with 10% phenol-free/growth factor reduced Matrigel (Corning Inc., Corning, NY). Cultures were treated with 1 μg/ml doxycycline from the beginning of organoid culture or only subsequent to passaging, as indicated. On day 9, cells were washed with cold PBS and pelleted. Organoids were dissociated with subsequent treatments of Dispase (1 U/ml) (Stem Cell Technologies, Vancouver, Canada) and TrypLE (Thermo Fisher Scientific, Waltham, MA). Cell pellets were then plated in a 24-well low adhesion dish in phenol-free tumor media supplemented with 5% phenol-free/growth factor reduced Matrigel. Images were collected on day 6 following passaging.

### Mice and tumor studies

**Fig. 1:**
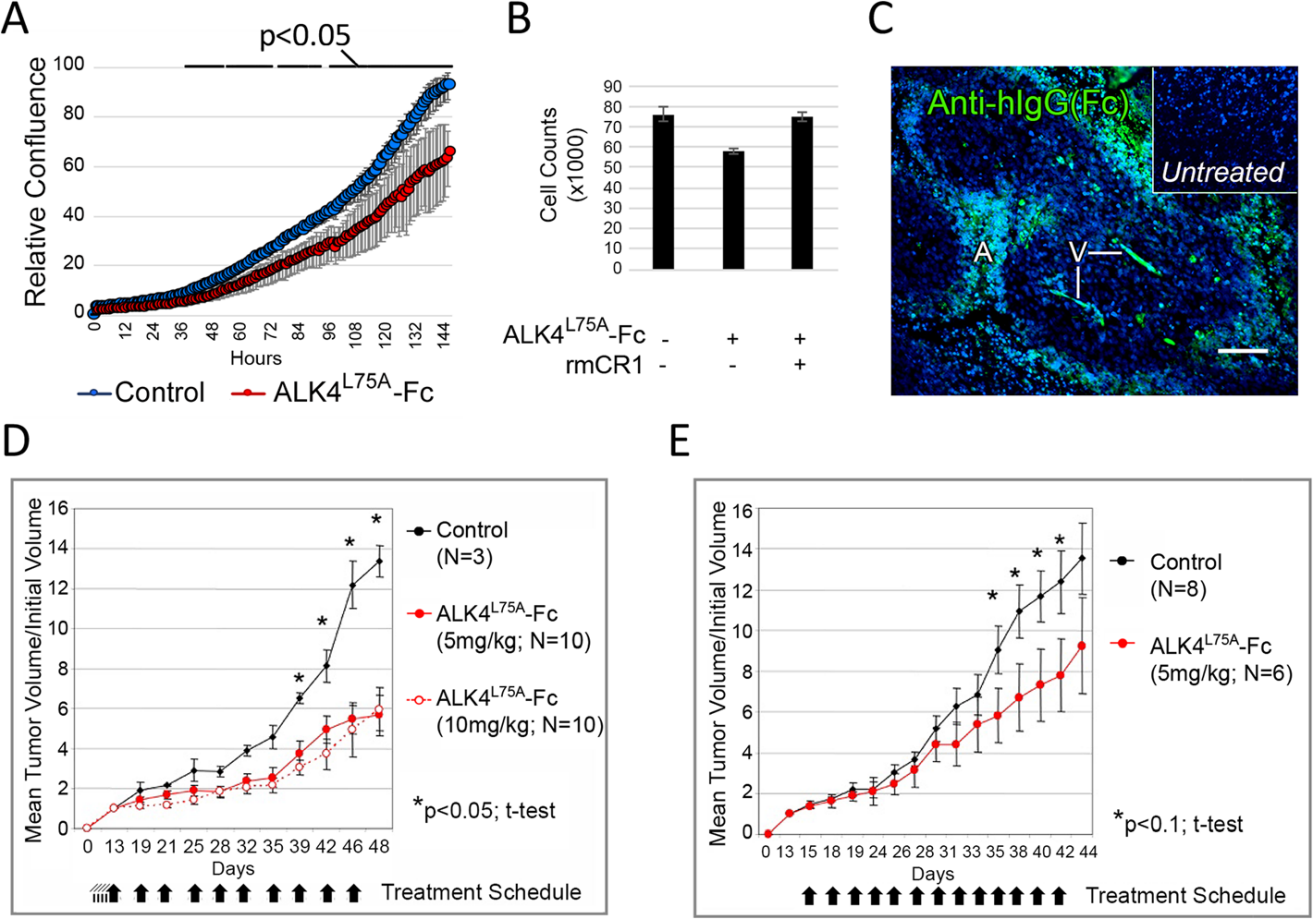
Recombinant purified ALK4^L75A^-Fc inhibits CRIPTO-dependent tumor cell growth in vitro and in vivo. **a** Confluency of NCCIT cells grown in vitro for 6 days in the presence (10 μg/ml) or absence of purified recombinant ALK4^L75A^-Fc. Standard deviation across triplicates is shown. **b** Effect of rmCRIPTO on the growth inhibitory effect of ALK4^L75A^-Fc in NCCIT cell cultures. Standard deviation across triplicates is shown. **c** Representative immunofluorescent anti-human IgG staining of tumor tissue from mice bearing NCCIT xenografts that were treated with ALK4^L75A^-Fc (5 mg/kg, I.P.) or vehicle (inset). A, acellular region; V, presumptive vasculature. Scale bar = 100 μm. **d**, **e** Effect of ALK4^L75A^-Fc injection compared to saline on growth of NCCIT xenografted tumors at the indicated doses and over the indicated time periods

**Fig. 2:**
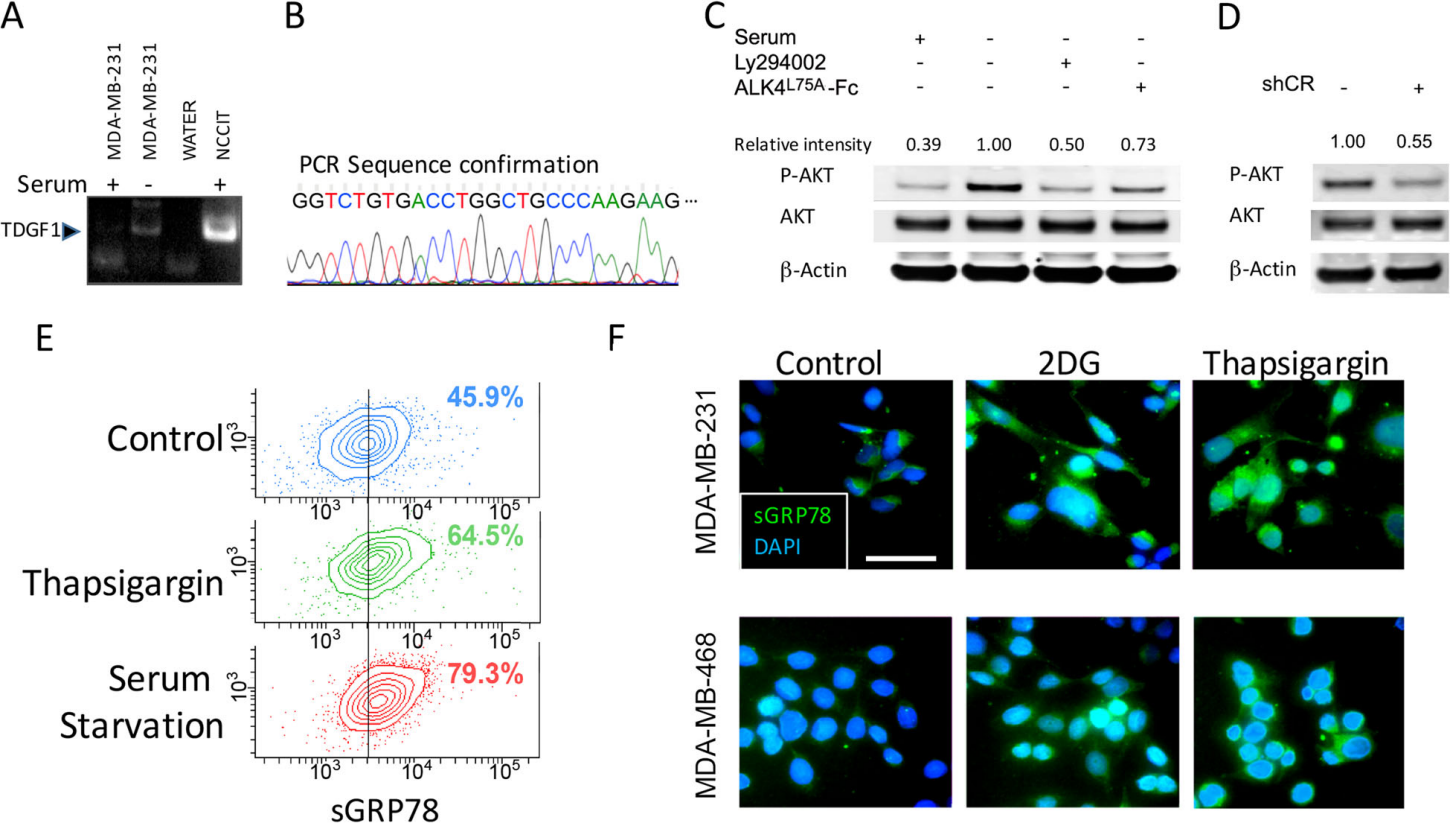
Stress response of the CRIPTO signaling pathway in breast cancer cell lines. **a** RT-PCR for TDGF1 (CRIPTO) in MDA-MB-231 under serum starvation. **b** Confirmation of TDGF1 sequence of the PCR product from **a**. **c** Western blot indicating that ALK4^L75A^-Fc blocks starvation-induced AKT activation in MDA-MB-231 cells. **d** AKT activation in MDA-MB-231 cells harboring a doxycycline-inducible shCRIPTO construct. **e** Flow cytometric analysis of surface GRP78 under thapsigargin treatment or serum starvation in MDA-MB-231. The percentage of cells brighter than the control midpoint are given from one of two independent experiments. **f** Immunofluorescent detection (prior to fixation) of surface GRP78 in 2-deoxyglucose (2-DG)- or thapsigargin-treated MDA-MB-231 and MDA-MB-468 cells. Representative images from one of three independent experiments are shown. Scale bar = 50 μm

Athymic female nude mice (for NCCIT grafts) and 10–12-week-old SCID beige female mice (for orthotopic breast cancer xenografts) were obtained from Charles River Laboratories (Wilmington, MA). All animal care and procedures were approved and monitored by an Institutional Animal Care and Use Committee. 1 × 10^6^ NCCIT cells (ATCC, Manassas, VA, CRL-2073) in phosphate-buffered saline were injected into each flank of nude mice and allowed to graft for 1 week (Fig. 1d) or 2 weeks (Fig. 1e) as indicated, prior to first treatment with soluble ALK4^L75A^-Fc or vehicle. As indicated, mice were treated with ALK4^L75A^-Fc daily for 5 days prior to overt tumor formation followed by bi-weekly injection or were treated bi-weekly after appearance of measurable tumor growth at 2 weeks. Tumor growth was measured with calipers. Cell suspensions of 1 × 10^5^ (Fig. 6a, c) or 2.5 × 10^5^ cells (Fig. 6b) for MDA-MB-231 and 1 × 10^4^ cells for MDA-MB-468 (Fig. 6e) in 40 μl of 50% Matrigel (50% media) were injected transdermally into the #4 fat pad of recipient mice, and tumors were allowed to form and grow to 1 cm or until mice were moribund or tissue was required for analysis. Following euthanasia, tumor tissue and other internal organs were harvested for processing. The lungs were isolated and imaged immediately on a M165 Fc fluorescent stereomicroscope (Leica Biosystems, Wetzlar, Germany) using Leica software. Metastases were identified as fluorescent green foci of varying sizes and were enumerated from static images. Mice were administered doxycycline ad libitum in drinking water (doxycycline hyclate, 0.8 mg/ml).

### Tumor sections

Freshly dissected tumor tissue was manually sectioned into slices < 4 mm in diameter and fixed overnight at 4 °C in 10% neutral buffered formalin. Fixed tissues were transferred to 70% ethanol for storage. Processing for histology followed standard protocols including paraffin embedding, sectioning at 5 μm thickness, baking for 1 h at 55 °C, and deparaffinizing with Histochoice™ Clearing agent (Amresco Inc., Solon, OH) or CitriSolv (Decon Labs, King of Prussia, PA) (Fig. 5e, Supplemental Fig. 3B only), and rehydration through graded alcohol/water washes. Antigen retrieval was achieved by boiling samples in citrate buffer (10 mM citric acid, 0.05% Tween 20, pH 6.0). Tumor sections were subjected to histological analysis and immunostaining with antibodies to E-cadherin (24E10, Cell Signaling Technology, Danvers, MA), MKI67 (SP6, Abcam, Cambridge, UK), HIF1α (S46-16, BioLegend, San Diego, CA), cleaved caspase-3 (5A1E, Cell Signaling Technology, Danvers, MA), CD31-PE (MEC13.3, BioLegend, San Diego, CA), GRP78 (IH11-1H7, Thermo Fisher Scientific, Waltham, MA), CRIPTO (ab19917, Abcam, Cambridge, UK), phalloidin (ab176757, Abcam, Cambridge, UK), donkey anti-rabbit IgG AlexaFluor 647 (ab150075, Abcam, Cambridge, UK), goat anti-mouse IgG PE/Cy5.5 (130784, Abcam, Cambridge, UK), and DAPI (Thermo Fisher Scientific, Waltham, MA). GRP78/CRIPTO/phalloidin samples were also blocked with mouse Fc receptor blocking reagent (Miltenyi Biotec, Bergisch Gladbach, Germany). Stained slides were imaged on a Nikon Eclipse Ni microscope with DSRi2 camera (Nikon, Tokyo, Japan) or an SP8 white light confocal microscope (Leica Biosystems, Wetzlar, Germany). For quantification of proliferation in stressed microenvironments, 12 high-powered images were captured from 7 mock and 5 ALK4^L75A^-Fc-expressing tumors by scanning for HIF1α positivity without other fluorescent channels visible. Images were subsequently captured across all relevant fluorescent channels to observe costaining with HIF1α and MKI67 antibodies (and DAPI). These were manually scored blind (i.e., unlabeled) and then assigned to their appropriate tumor type, post hoc. Pimonidazole was injected intraperitoneally at 60 mg/kg according to the recommendations in the Hypoxiprobe™ kit (Hypoxyprobe, Burlington, MA) and detection of signal was carried out according to the manufacturer’s recommendations.

### Western blots

Protein lysates from cultured cells and immune precipitates from conditioned media were suspended in RIPA buffer (50 mM Tris, pH 7.4, 1% Nonidet P-40 Substitute (Sigma-Aldrich, St. Louis, MO), 0.5% sodium deoxycholate, 0.1% SDS supplemented with protease inhibitor cocktail (Sigma-Aldrich, St. Louis, MO #P8340), and phosphatase inhibitor cocktail (Thermo Fisher Scientific, Waltham, MA, #78420)). Samples were boiled in LDS loading buffer prior to loading and were run on pre-cast 4–12% gradient Bis-Tris, 1.5 mm × 15 well gels (Invitrogen, Carlsbad, CA, #NP0336BOX). Proteins were transferred on an iBlot2 Dry Blotting System (Thermo Fisher Scientific, Waltham, MA) to PVDF (Invitrogen, Carlsbad, CA, #IB24002) or nitrocellulose (Invitrogen, Carlsbad, CA, #IB23002) membranes for protein detection. Membranes were blocked in Intercept Blocking buffer (Li-Cor Biosciences, Lincoln, NE) and probed with the following: mouse anti-human AKT (40D4, Cell Signaling Technology, Danvers, MA #2920, 1:2000), rabbit anti-human phospho-AKT (Ser473) (D9E, Cell Signaling Technology, Danvers, MA #4060, 1:2000), and rat anti-human β-actin (W16197A, BioLegend #664802, 1:10,000). The following secondary antibodies were used to detect unconjugated primary antibodies: goat anti-mouse IgG H&L, AF790 (Thermo Fisher Scientific, Waltham, MA, #A11375, 1:20,000); goat anti-rabbit IgG H&L, AF680 (Thermo Fisher Scientific, Waltham, MA, #A21076, 1:1000); and donkey anti-rat IgG H&L, Dylight 800 (Thermo Fisher Scientific, Waltham, MA, #SA5-10032, 1:10,000).

### RT-PCR

Mouse and human TDGF1 (forward: TCCTTTTGTGCCTGCCCTC; reverse: CACAGGGAACACTTCTTGG) or commercially available primers for ACTB (Integrated DNA Technologies, Coralville, IA, IDT-PrimeTime Std qPCR Assay Hs.PT.39a.22214847) were included in standard PCR or quantitative real-time RT-PCR using the Power SYBR Green Cells-to-CT kit (Thermo Fisher Scientific, Waltham, MA, #4402954) according to the manufacturer’s instructions. Real-time PCR was carried out on a lightCycler 480 (Roche, Basel, Switzerland), and comparisons were made using the ΔΔCt method with β-actin as a control transcript.

### RNA sequencing

A mouse bearing one pair of contralateral LV-S14-Mock and LV-S14-ALK4^L75A^-Fc tumors of approximately equal size (*D* = ~ 0.66 cm) was administered doxycycline ad libitum in water for 96 h prior to tumor harvest. We generated sequencing libraries from several snap frozen, cold pulverized 2-mm fragments of tumors corresponding to a spherical wedge of approximately 1/3 of the total tumor volume and extending from the center to the periphery immediately after isolation. We employed Truseq™ RNA preparation kits (Illumina, San Diego, CA), according to the manufacturer’s instructions. Libraries were validated using an Agilent Technologies bioanalyzer 2100 and Quant-iT™ HS dsDNA assay (Life Technologies, Carlsbad, CA). One hundred base, paired end sequencing was carried out on a Hiseq2000 (Illumina, San Diego, CA). Reads were mapped to the human genome (hg38) using STAR [37]. We quantified gene expression as FPKM (fragments per kilobase of gene model per million reads) using Cufflinks [38].

## Results

### ALK4^L75A^-Fc treatment inhibits CRIPTO-dependent tumor cell growth in vitro and in vivo

Embryonal carcinoma-derived NCCIT cells provide a model tumor cell system to test CRIPTO inhibitors as these cells have stem cell properties and express high levels of CRIPTO protein. We previously reported that growth of NCCIT cells in vitro is inhibited by CRIPTO knockdown or treatment with a neutralizing GRP78 antibody that disrupts CRIPTO/GRP78 binding [26]. Here, we show that treatment of NCCIT cells with ALK4^L75A^-Fc similarly inhibits NCCIT cell growth (Fig. 1a) and that this effect is rescued by addition of soluble recombinant CRIPTO protein (Fig. 1b). Next, we sought to test the effects of ALK4^L75A^-Fc protein on NCCIT xenografts grown in the flanks of nude mice by injecting the reagent intraperitoneally. ALK4^L75A^-Fc accumulated in NCCIT tumors since high immuno-reactivity was visible in occasional cells distributed throughout the tumors, in structures resembling vessels (V) and in cells distal to the presumptive vasculature at the boundary between intact tumor tissue and acellular zones (A) (Fig. 1c, Supplemental Fig. 3). Critically, treating mice with ALK4^L75A^-Fc slowed the growth of these very aggressive NCCIT cell tumors when injected either before (Fig. 1d) or after (Fig. 1e) measurable tumors were detectable. No adverse effects were observed when mice were injected with ALK4^L75A^-Fc at the highest dose tested of 10 mg/kg (Fig. 1d, and data not shown).

### CRIPTO signaling is regulated by stress in breast cancer cells

MDA-MB-231 is an aggressive, poorly differentiated, TNBC cell line that has been reported to express CRIPTO [39]. While we found that expression of CRIPTO transcripts was barely detectable in these cells under standard growth conditions, CRIPTO levels increased when these cells were grown overnight in medium that had reduced glucose levels and lacked serum-derived growth factors (Opti-MEM, GIBCO) (Fig. 2a, b). Culturing MDA-MB-231 cells under these conditions also resulted in increased levels of phospho-AKT (Ser473), which could be inhibited by treating cells with ALK4^L75A^-Fc or the PI3K-inhibitor LY294002 (Fig. 2c). Expression of a short hairpin targeting *CRIPTO* similarly reduced AKT phosphorylation in MDA-MB-231 cells (Fig. 2d, Supplemental Fig. 1). Cell surface GRP78 levels also increased under these growth conditions to an even greater extent than was observed following treatment with thapsigargin, which is known to strongly increase the expression and cell surface localization of GRP78 (Fig. 2e). Finally, and consistent with previous results [40], both thapsigargin and the glycolysis inhibitor 2-deoxyglucose (2-DG) increased cell surface GRP78 levels relative to untreated controls in both MDA-MB-231 cells and a second TNBC cell line, MDA-MB-468 (Fig. 2f). Together, these results are consistent with CRIPTO/GRP78 signaling being stress responsive in breast cancer cells.

### ALK4^L75A^-Fc inhibits adaptation of breast cancer cells to glycolytic stress

**Fig. 3:**
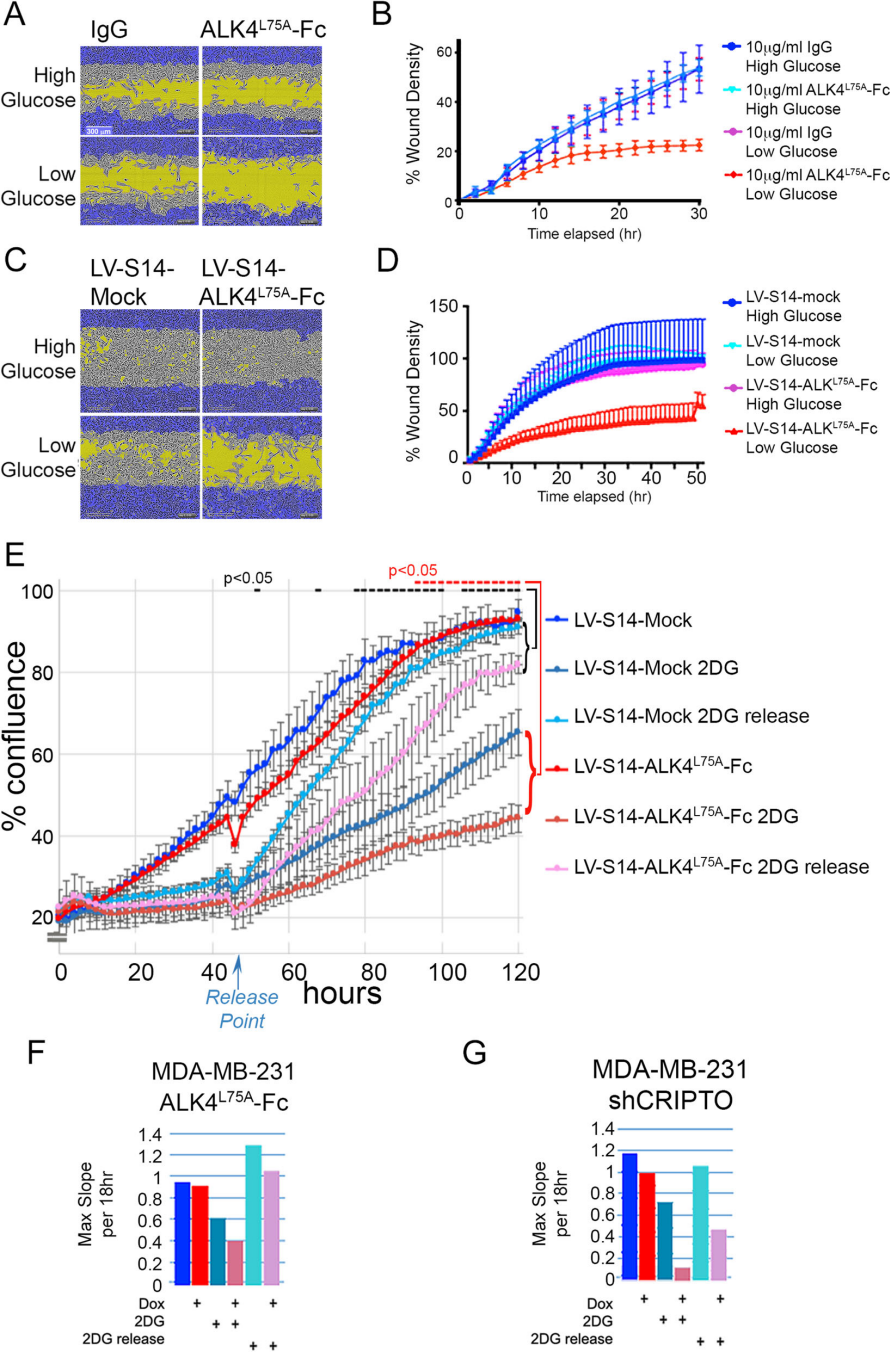
ALK4^L75A^-Fc reduces migration and proliferation in stressed breast cancer cells, Scale bar = 300m. **a**, **b** In vitro scratch wound filling by MDA-MB-231 cells treated with soluble ALK4^L75A^-Fc protein under glucose restriction. Representative images from among 5 replicates per treatment are shown. **c**, **d** In vitro scratch wound filling of ALK4^L75A^-Fc-expressing and mock, LVS14-transduced MDA-MB-231 cell cultures under glucose restriction. Representative images from among 5 replicates per treatment are shown. **e** ALK4^L75A^-Fc inhibits MDA-MB-231 human breast cancer cells’ growth recovery from 2-DG treatment. Replicates = 3 *Student’s *t* test. **f** Maximum slope values from the graph in **e**. **g** Growth rate of MDA-MB-231 cells expressing shCRIPTO under 2-DG challenge and release, represented as maximum slope. All time-lapse studies were conducted with 3–5 replicates and were repeated 2–3 times

Next, we tested the effect of ALK4^L75A^-Fc on cellular phenotypes related to EMT and stemness in the context of stress adaptation in these TNBC cell lines. Notably, treatment of MDA-MB-231 cells with ALK4^L75A^-Fc had little effect on cell migration in standard, high glucose culture conditions (4.5 g/l), but inhibited migration under conditions of glucose deprivation (1 g/l) (Fig. 3a, b). In order to provide an alternative method of delivering ALK4^L75A^-Fc and facilitate parallel in vitro and in vivo studies, we generated a lentiviral vector (LV-S14) that allows doxycycline (Dox)-inducible expression of secreted ALK4^L75A^-Fc while also providing luminescent and fluorescent tracking capability (Supplemental Fig. 1). The effect of ALK4^L75A^-Fc using this delivery method was highly similar to that resulting from treatment of MDA-MB-231 cells with purified ALK4^L75A^-Fc protein. Thus, Dox treatment of LV-ALK4^L75A^-Fc MDA-MB-231 cells caused reduced migration specifically under low glucose conditions while Dox treatment of LV-Mock MDA-MB-231 (negative control) had no effect (Fig. 3c, d). Similarly, Dox-induced ALK4^L75A^-Fc had little effect on the overall proliferation of MDA-MB-231 cells in standard (high glucose) media but increased sensitivity to growth inhibition by 2-DG (Fig. 3e, f). Strikingly, when 2-DG was removed from the media after 48 h, control (mock) cells grew faster than ALK4^L75A^-Fc-expressing cells and untreated control cells, consistent with a role for CRIPTO in adaptive cellular growth responses under glucose deprivation in vitro (Fig. 3e, f). Targeting CRIPTO expression with a Dox-inducible shRNA similarly reduced the rate of growth of MDA-MB-231 cells challenged by or released from 2-DG treatment (Fig. 3g).

**Fig. 4:**
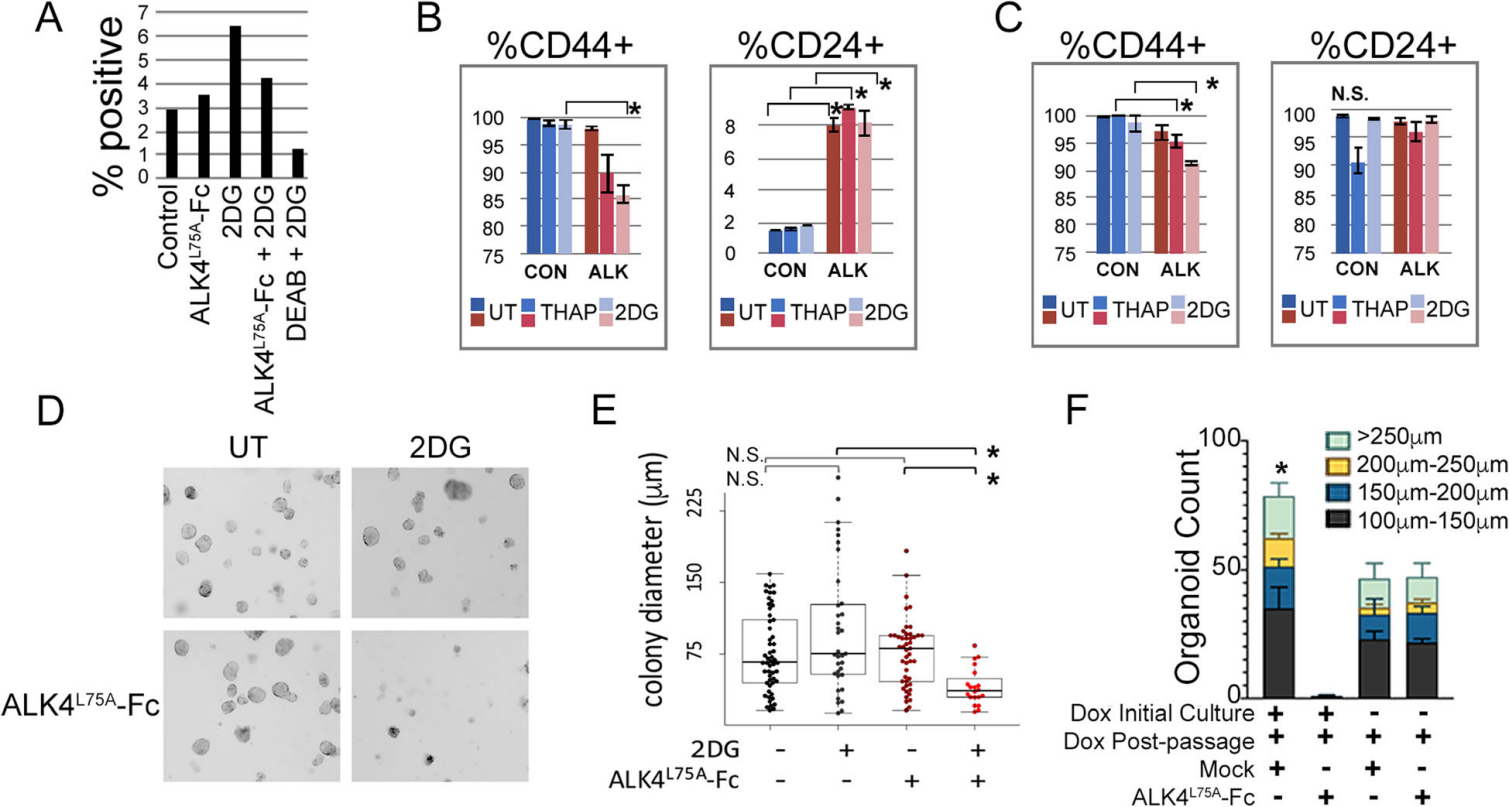
The CRIPTO blocking agent, ALK4^L75A^-Fc, reduces cancer stem cell properties in stressed breast cancer cells. **a** Stress and ALK4^L75A^-Fc associated changes in Aldefluor markers. **b**, **c** Effects of ALK4^L75A^-Fc on CD44 expression in thapsigargin- or 2-DG-treated MDA-MB-231 (**b**) and MDA-MB-468 cells (**c**). *Student’s *t* test, *p* < 0.05. **d** Changes in suspension colony frequency and growth of LVS14-Mock- and LVS14-ALK^L75A^-Fc-transduced MDA-MB-468 cells following stress. Duplicate wells were examined, and the experiment was carried out twice. **e** Quantification of suspension colony growth from the experiment shown in **d** and using multiple images. *Fisher’s exact test, *p* < 0.05. **f** Serial passage rates of MDA-MB-468 cells (in triplicate) induced to express ALK^L75A^-Fc. *Kruskal-Wallis, *p* = 0.0152

ALK4^L75A^-Fc also inhibited properties associated with cancer stem cells (CSC) and breast cancer cell aggressiveness in the context of experimental stress (Fig. 4). For example, 2-DG increased the percentage of cultured MDA-MB-231 cells that were positive for the stem cell marker Aldefluor and this effect was inhibited by treatment with ALK4^L75A^-Fc (Fig. 4a). Similarly, ALK4^L75A^-Fc reduced cell surface levels of the cancer stem cell-associated marker CD44 in MDA-MB-231 and MDA-MB-468 cells treated with 2-DG or thapsigargin, while increasing expression of the CSC contraindicator CD24 in MDA-MB-231 cells (Fig. 4b, c) [41]. MDA-MB-468 cells express naturally high levels of CD24 even prior to treatment (Fig. 4c). Colony growth under non-adherent conditions, such as methylcellulose and Matrigel cultures, provides a measure of cancer stem cell function and aggressiveness. MDA-MB-468 cells formed discrete colonies in methylcellulose, and the size of these colonies was not significantly altered by 2-DG treatment, although some large colonies were noted in 2-DG-treated cultures (Fig. 4d, e). Whereas ALK4^L75A^-Fc treatment did not affect the size of colonies grown in the absence of 2-DG, it strongly inhibited colony growth in the presence of 2-DG (Fig. 4d, e). We also examined the growth of colonies from ALK4^L75A^-Fc- and mock-transduced MDA-MB-468 cells plated in organoid culture conditions containing Matrigel. Similar to what was observed in methyl-cellulose cultures (Fig. 4d, e), Dox induction of ALK4^L75A^-Fc in MDA-MD-468 cells grown in organoid culturing conditions had no effect on initial organoid growth but resulted in almost no organoids in secondary cultures compared to uninduced primary cultures and mock controls, which produced many secondary organoids (Fig. 4f, Supplemental Fig. 2). These results suggest that the stress of 2-DG and reseeding renders MDA-MB-468 cells sensitive to CRIPTO blockade by ALK4^L75A^-Fc.

### CRIPTO antagonism with ALK4^L75A^-Fc alters breast cancer stem cell, EMT, and stress response properties in vivo

**Fig. 5:**
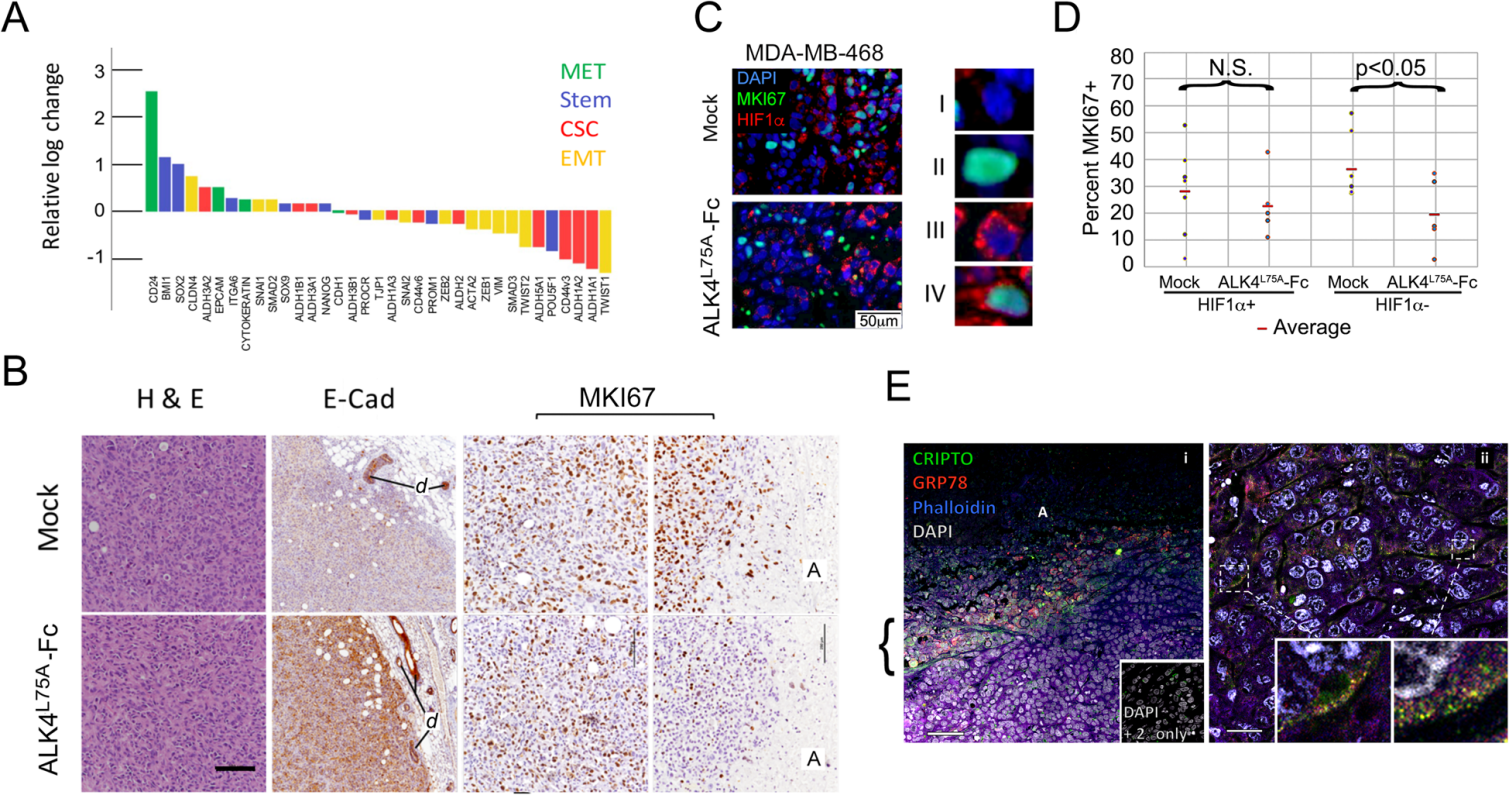
ALK4^L75A^-Fc reduces aggressive breast cancer cell phenotypes and proliferation of stressed cancer cells in vivo. **a** Expression changes in select stem cell, cancer stem cell, and EMT/MET associated transcripts in a contralateral pair of mock and ALK4^L75A^-Fc MDA-MB-231 tumors following 96 h of in vivo doxycycline treatment. **b** Representative images of mock and ALK4^L75A^-Fc-expressing MDA-MB-231 xenografts (*n* = 3 of each type). H&E, hematoxylin/eosin staining. E-Cadherin at the periphery of advanced tumors (2nd column). d = mammary duct. MKI67 staining of tumors in highly cellular areas and adjacent to acellular regions, A (right 4 panels). Scale = 250 μM. **c** Representative images of the overlap of MKI67 (green) and HIF1a (red) staining in MDA-MB-468 tumor sections with and without ALK4^L75A^-Fc expression. Four classes of cells could be identified, I–IV. **d** Blinded quantification of the four cell classes indicated in **c** across a set of 12 images (7 mock and 5 ALK4^L75A^-Fc) collected from 2 MDA-MB-468 mock and 2 MDA-MB-468 ALK4^L75A^-Fc tumors. *p < 0.05; Student’s *t* test. **e** Representative (*n* = 2) costaining for CRIPTO (green), GRP78 (red), phalloidin (blue), and DAPI (gray). Bracket = acellular region-adjacent CRIPTO staining cells. Scale bars = 100 μM (i) and 25 μM (ii), insets show colocalization of CRIPTO and GRP78 (yellow) at the cell periphery in bracketed regions

Next, we examined the effect of ALK4^L75A^-Fc on breast cancer growth and metastasis in vivo. We transplanted stably transduced LV-S14-Mock and LV-S14-ALK4^L75A^-Fc MDA-MB-231 cells orthotopically into mammary fat pads of SCID mice, allowed tumors to grow to a size of 60–70 mm in diameter, and then administered Dox for 96 h to induce ALK4^L75A^-Fc. We compared the expression patterns of several previously reported EMT and stem cell markers (Fig. 5a). Expression levels of known cancer stem cell markers declined under ALK4^L75A^-Fc treatment, including the majority of aldehyde dehydrogenase (ALDH) transcripts that have been shown to contribute to positivity for the cancer stem cell-associated Aldefluor reagent and the CSC-associated isoforms of CD44 (CD44v3, CD44v6) [41, 42]. Markers associated with normal stem cells such as POU5F1, SOX2, and NANOG were mixed in the directionality of their change. The majority of transcripts for known EMT markers (e.g., VIM, ACTA2, ZEB1, ZEB2, SNAI2, TWIST1, TWIST2) also declined under ALK4^L75A^-Fc induction although SNAI1 was slightly elevated (Fig. 5a). Conversely, markers of epithelial fate and mesenchymal to epithelial transition (MET) (e.g., EPCAM, Cytokeratins (avg.), CLDN4, and the MaSC-associated marker CD24 (also a putative CSC contraindicator)) increased under ALK4^L75A^-Fc treatment (Fig. 5a). Together, these data suggest that ALK4^L75A^-Fc promotes a transition from a stem cell-like, mesenchymal phenotype to a more differentiated, epithelial phenotype in vivo.

We did not observe major morphological changes in MDA-MB-231 tumor cells following 96 h of ALK4^L75A^-Fc induction in vivo (data not shown). However, examination of a contralateral pair of tumors induced with Dox for 96 h suggested early gene expression changes toward a more epithelial, less mesenchymal, less CSC-like state when comparing the ALK4^L75A^-Fc-expressing tumor to its mock counterpart (Fig. 5a). Nevertheless, even when tumor cells were induced to express ALK4^L75A^-Fc for 6 weeks, few differences in histopathological features were noted (Fig. 5b, left panels). Both treated and untreated tumors retained an overall carcinomatous histopathology and had regions of apparent necrosis surrounded by regions of high cellularity (see also Supplemental Fig. 3). However, in some regions, ALK4^L75A^-Fc-expressing cells were slightly larger than their mock-transduced counterparts (Mock 2-4 rbc vs ALK4^L75A^-Fc 6-8 rbc) (Fig. 5b and data not shown). We noted very few pyknotic nuclei in the highly cellular zones of either mock or ALK4^L75A^-Fc-expressing tumors. Consistent with this, the highly cellular regions of both mock and ALK4^L75A^-Fc tumors only rarely showed cells positive for the apoptosis marker cleaved caspase 3, though the boundary with acellular zones was strongly positive for this marker in both cases (Supplemental Fig. 3). Though treated and untreated tumor cells appeared grossly similar and E-cadherin transcript levels (CDH1) did not appear to be acutely modulated by ALK4^L75A^-Fc in MDA-MB-231 xenografts (Fig. 5a), end stage tumors expressing ALK4^L75A^-Fc stained more strongly for E-cadherin protein (compared for instance to endogenous mammary ducts (d)), consistent with ongoing reversal of EMT and promotion of a more differentiated phenotype over the longer term (Fig. 5b).

Although we found that ALK4^L75A^-Fc expression had no obvious effect on cellular proliferation rates in highly cellular regions of tumors as judged by mitotic counts (not shown) and MKI67 positivity (Fig. 5b, right panels), ALK^L75A^-Fc-expressing tumors showed occasional abnormal mitoses and a marked reduction in MKI67 staining specifically in regions adjacent to necrotic areas when compared to mock controls (Fig. 5b, far right panels). Acellular regions in tumors frequently represent avascular regions where nutrient supply is insufficient to support cellular survival, and the cells adjacent to these regions are expected to be under nutrient deprivation stress. Indeed, we found regions adjacent to these acellular zones to stain positive for pimonidazole adducts indicating that they are hypoxic (Supplemental Fig. 3). Therefore, we quantified Ki67 positivity in cells exhibiting HIF1α stabilization to compare proliferation rates of stressed cells in tissue sections from control and ALK4^L75A^-Fc-expressing tumors (Fig. 5c, d). We were able to identify all four classes (I–IV) of DAPI-stained nuclei in tissue sections from LV-S14-Mock and LV-S14-ALK4^L75A^-Fc tumors (Fig. 5c). Enumeration of these four classes across multiple micrographs per tumor type revealed a statistically significant reduction in double-positive (MKI67+,HIF1a+) cells in ALK4^L75A^-Fc-expressing tumors (Fig. 5d). Consistent with our observations in vitro, these in vivo data indicate that CRIPTO signaling is required for tumor cell proliferation under conditions of stress and that this stress-adaptation signaling can be blocked with ALK4^L75A^-Fc. Indeed, immune detection of CRIPTO and GRP78 in tumor xenografts showed robust staining for both CRIPTO and GRP78 at the cell periphery specifically in regions we previously identified as nutrient deprived (Fig. 5e, Supplemental Fig. 3). CRIPTO and GRP78 were often colocalized at the cell surface in these regions (Fig. 5e, ii insets). These regions also exhibited qualitative differences in phospho-SMAD2/3 and phospho-AKT staining (Supplemental Fig. 4).

### CRIPTO blockade with ALK4^L75A^-Fc inhibits growth and metastasis of breast cancer xenografts

**Fig. 6:**
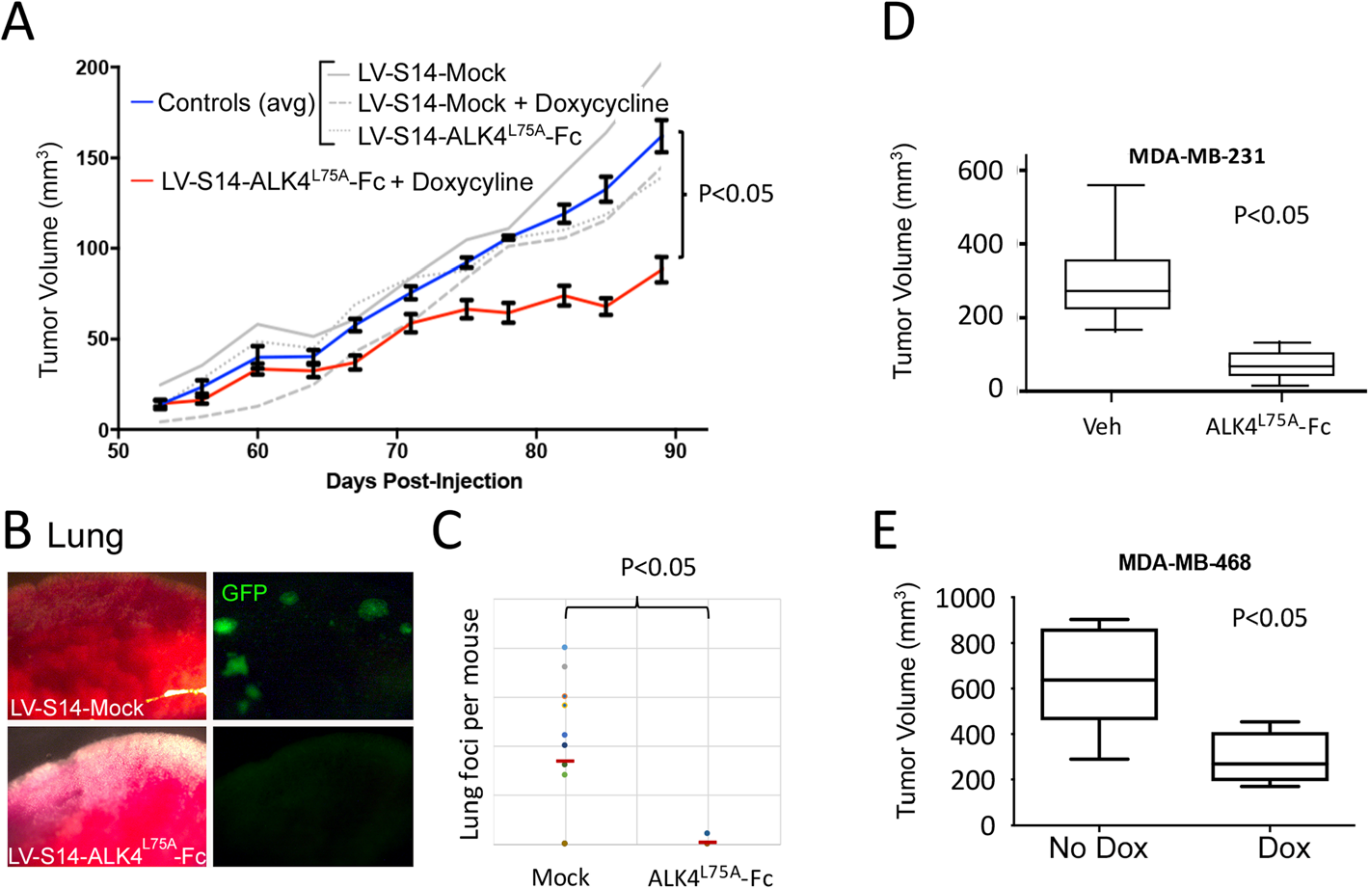
Expression of ALK4^L75A^-Fc in vivo inhibits growth and metastasis of breast cancer xenografts. **a** Orthotopic MDA-MB-231 xenograft growth in the presence or absence of doxycycline induction of ALK4^L75A^-Fc. Student’s *t* test, *p* < 0.05 (across each of the final four measurements). *n* = 15 per group. **b** Bright field and fluorescent images of representative lung tissue samples from MDA-MB-231 xenografted mice in the absence (LV-S14-Mock; *n* = 12) or presence (LV-S14-ALK4^L75A^-Fc; *n* = 8) of ALK4^L75A^-Fc expression. **c** Number of macroscopic fluorescent metastatic foci in the lungs of mice bearing control or ALK4^L75A^-Fc-expressing tumor cells. Fisher’s exact test. **d** Size of primary MDA-MB-231 orthotopic xenografts at 5 weeks post-inoculation in mice injected intraperitoneally with ALK4^L75A^-Fc (10 mg/kg; *n* = 6) or vehicle (*n* = 4). **e** Size of MDA-MB-468 orthotopic xenografts at 5 weeks in the presence (*n* = 6) or absence (*n* = 4) of ALK4^L75A^-Fc expression

We conducted a larger tumor study to examine the overall impact of ALK4^L75A^-Fc on breast cancer growth and spread. LV-S14-Mock and LV-S14-ALK4^L75A^-Fc MDA-MB-231 cells were transplanted orthotopically into nude mice, and then, Dox was administered 2 weeks later to induce expression of ALK4^L75A^-Fc. Tumors arising from cells that carry Dox-inducible ALK4^L75A^-Fc grew more slowly than those arising from LV-S14-Mock cells or with either cell type in the absence of Dox (Fig. 6a). Furthermore, at the endpoint of the study, gross fluorescent visualization of lung tissue from mice with MDA-MB-231 tumors lacking ALK4^L75A^-Fc expression revealed numerous macro- and micro-metastatic lesions (Fig. 6b). By contrast, of the 8 mice with ALK4^L75A^-Fc-expressing tumors, only 1 with a single macro-metastasis was observed (Fig. 6c). Critically, these anticancer effects of ALK4^L75A^-Fc were not restricted to overexpression of ALK4^L75A^-Fc in MDA-MB-231 cells since injection of purified ALK4^L75A^-Fc (10 mg/kg; I.P.) also inhibited MDA-MB-231 tumor growth (Fig. 6d), and similar to what we observed in MDA-MB-231 cells, ALK4^L75A^-Fc induction in a second TNBC model (MDA-MB-468 cells) following transplantation also inhibited growth of resulting tumors (Fig. 6e).

## Discussion

Phenotypic plasticity allows cells to adapt to stresses such as nutrient deprivation, hypoxia, and chemotherapy and likely represents a key contributor to tumor cell heterogeneity and the major clinical problems of dormancy, metastasis, therapy resistance, and recurrence. Our studies indicate that CRIPTO plays critical roles in plasticity and the adaptive process. We show that the CRIPTO directed antagonist ALK4^L75A^-Fc is active against poorly differentiated TNBC cells in vitro and in vivo, and that it blocks their ability to adapt to stresses such as nutrient deprivation that are commonly encountered by tumors. Specifically, ALK4^L75A^-Fc decreased the expression of EMT and CSC markers that are hallmarks of cancer aggressiveness, blocked proliferation of cells in stressed microenvironments in vivo, and also reduced tumor burden especially at metastatic sites.

We focused on two models of TNBC due to the unmet need for molecular targets in this subtype of breast cancer and because of their tractability as tumor xenografts for modeling tumor growth and metastasis. Our study establishes proof of principal for ALK4^L75A^-Fc in treating these tumors. It remains to be determined whether increases in surface CRIPTO or surface GRP78 are more important in rendering the CRIPTO/GRP78 signaling axis sensitive to stress. However, we note that CRIPTO is overexpressed in a range of cancers including over 80% of breast cancers, and accordingly, some preliminary studies have indicated CRIPTO effects in both ER+ and Her2+ breast cancers [18]. Using similar approaches to those described here, it should be possible to identify additional tumor types susceptible to ALK4^L75A^-Fc using patient-derived organoid and xenograft models (PDO and PDX, respectively) derived from diverse breast cancer patients and representing the broader intertumoral heterogeneity of breast cancer.

CRIPTO’s effects on breast cancer cells are likely mediated by its joint modulation of the TGF-β pathway and growth factor-like signaling (i.e., activation of SRC/MAPK/PI3K pathways), and there may also be important roles for other pathways regulated by CRIPTO including WNT and NOTCH [33, 34]. We previously showed that ALK4^L75A^-Fc acts as a “ligand trap” that inhibits CRIPTO growth factor-like signaling, including AKT activation, in primary mouse mammary epithelial cells and a non-transformed human mammary epithelial cell line (MCF10A) [12]. The AKT pathway is a critical regulator of tumor cell growth and stem cell-like properties, and our results suggest that blocking this aspect of CRIPTO signaling plays a key role in inhibiting maintenance of the stem cell phenotype and oncogenesis. While there currently is no evidence supporting such a role, it remains formally possible that ALK4^L75A^-Fc also has additional unanticipated targets besides CRIPTO that contribute to its anti-tumor effects.

**Fig. 7:**
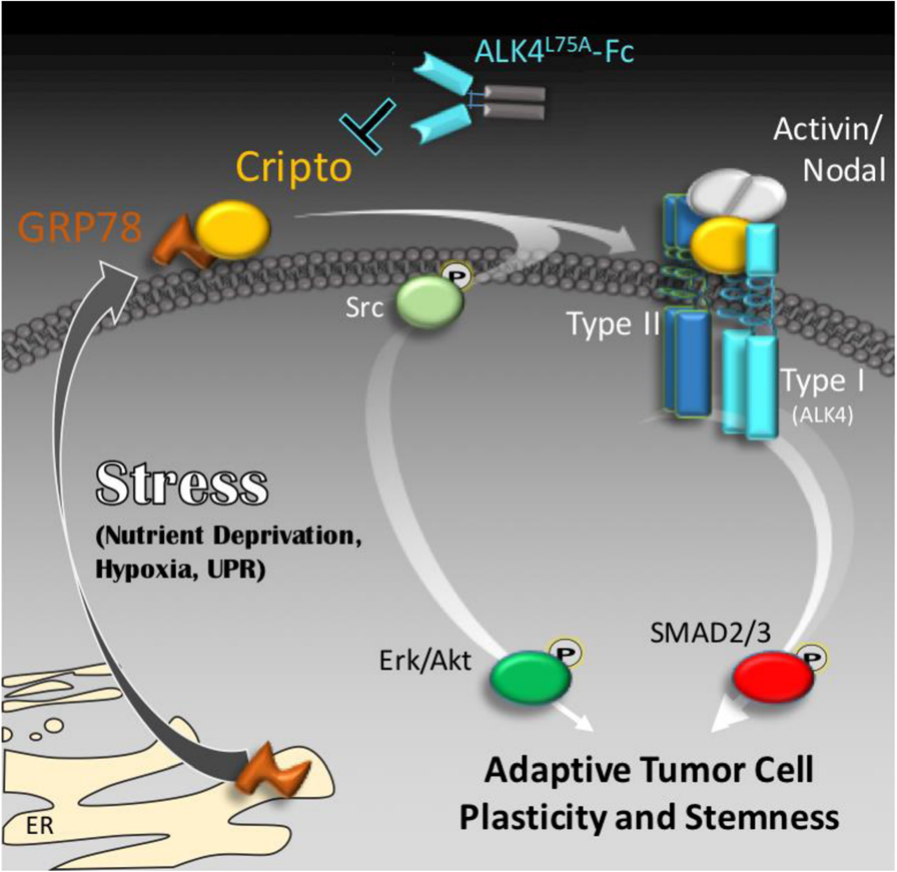
Graphical rendering of GRP78-dependent CRIPTO signaling driving stem-like tumor cell phenotypes. Cellular stresses that promote GRP78 re-localization to the cell surface could render such cells susceptible to CRIPTO-mediated signaling that balances growth factor-like signaling with TGF-beta pathway signaling to promote growth and plasticity

CRIPTO binding to GRP78 at the cell surface is thought to be critical for CRIPTO signaling function [12, 26, 27]. Although GRP78’s best defined roles in cellular stress responses involve its ER/UPR activities, it is now widely recognized that a portion of GRP78 re-localizes to non-ER locations including the cell surface in response to a variety of cellular stresses such as ER stress and nutrient deprivation. Cell surface GRP78 is proposed to mediate additional cellular stress responses such as those demonstrated for CRIPTO previously and in the present study [5, 29]. Notably, nutrient stress was reported to promote GRP78-dependent AKT phosphorylation in a variety of tumor cell lines including breast cancer cells [43]. Recently, GRP78 was also implicated in the maintenance/reestablishment of MEK/ERK signaling in therapy-resistant BRAF mutant melanomas, and in maintaining stemness in pancreatic cancer cells in response to therapy-associated oxidative and protein stress [44, 45]. Similarly, another recent report has indicted that cell surface GRP78 also marks cells with enhanced tumor- and metastasis-initiating capacities in breast cancer [46]. Based on the findings presented here, we propose that re-localization of GRP78 to the cell surface during stressful conditions enhances CRIPTO signaling and thereby promotes cellular plasticity, adaptation to stress, and tumor progression (Fig. 7).

Here, we specifically examined the effects of ALK4^L75A^-Fc under conditions of growth factor restriction, glucose deprivation, and ER stress, challenges that are regularly encountered by tumor cells in vivo*.* Although the effects of chemotherapeutic and molecularly targeted therapies were not examined in this study, these treatments elicit similar stresses to those examined here. Our in vitro studies indicate that while blocking CRIPTO/GRP78 signaling does not inhibit oncogenic behaviors such as cell migration in the absence of stresses such as nutrient deprivation, it sustains these behaviors in the presence of such stresses. Similarly, we find that targeting CRIPTO signaling with ALK4^L75A^-Fc specifically impacts cell proliferation in stressed microenvironments in tumors and inhibits metastasis, a process that requires tumor cells to adapt to multiple novel and stressful environments. Thus, cellular dependencies on CRIPTO and perhaps other stem cell/plasticity promoting pathways are likely to be dependent on contexts that call for cellular adaptation, including therapy response.

We have shown that in addition to compromising cellular stress adaptation, blocking CRIPTO signaling inhibits EMT and CSC phenotypes, the acquisition of which are both plastic/reversible processes [12]. EMT is associated with invasive phenotypes that may contribute to tumor cell dissemination, and its reversibility is critical for metastatic outgrowth [47]. The EMT phenotype has been shown to emerge in tumor cells under therapeutic challenge and to engender drug resistance [48]. Similarly, CSC activity (i.e., enhanced tumor propagating capacity of subpopulations of tumor cells) has been shown to be a metastable phenotype associated with specific biomarkers in breast and other cancers [49, 50]. Importantly, breast CSC phenotypes may be augmented by microenvironmental stresses [51]. In a notable example of this, Wicha and colleagues showed that certain stem cell populations (i.e., ALDH+ cells) reside in stressed tumor microdomains and increase following antiangiogenic, hypoxia-inducing therapy (Avastin) [52]. It remains to be determined if other critical cell processes like metabolism or trafficking, which may also be under CRIPTO control [53, 54], are critical contributors to overall cellular plasticity and adaptability.

## Conclusions

We conclude that TNBCs, and possibly many other types of cancers, critically rely on stress adaptive programs involving the CRIPTO/GRP78 signaling axis and that these can be inhibited by ALK4^L75A^-Fc. In a wide variety of cancers including breast cancers, subpopulations of cells exhibit specialized tumorigenic properties that contribute to disease progression and therapy resistance [55]. Elucidation of mechanisms governing tumor cell adaptation to stress has led to the identification of therapeutic targets such as CRIPTO for inhibiting plasticity and the emergence of subpopulations of cancer cells with facultative stem cell-like properties. While reagents such as ALK4^L75A^-Fc that block the function of these targets might show modest effects as single agents in some tumor types where many cells have secured ample nutrient supply, they are predicted to complement cytostatic and cytotoxic treatments by undercutting cells surviving debulking therapies that are capable of adapting and emerging as therapy-resistant clones following chemotherapy, recurring after dormancy, and disseminating through novel stress-inducing environments during metastasis.

## Supplementary Information

**Additional file 1: Supplemental Fig. 1.** A) Schematic of a lentiviral expression vector (S14) with fluorescent luminescent markers and doxycycline-inducible ALK4^L75A^-Fc. (B,C) Western blot demonstrating doxycycline-dependent expression of FLAG-tagged ALK4^L75A^-Fc in S14-transduced MDA-MB-231 cells, B and secretion into conditioned media, C. (D) Amplification curves for TDGF1 (ie CRIPTO) from equivalent starting material in MDA-MB231 cells transduced with a Dox dependent shCRIPTO vector. **Supplemental Fig. 2.** Morphology of organoids seeded into secondary cultures following treatment with Dox to induce ALK4^L75A^-Fc expression during primary organoid out growth. **Supplemental Fig. 3.** A) a panoramic view of a hematoxylin/Eosin stained MDA-MB-468 tumor section by which position and morphology can be used to assign regional and structural information. P=periphery, v=presumptive vasculature, C=cellular region, S =stressed zone (see B), A = acellular zone. No gross morphological differences apart from average size were noted for ALK4^L75A^-Fc expressing tumors relative to mock controls. (B) High magnification images of presumptive vasculature in MDA-MB-468 xenografts containing obvious red blood cells (left panels). Lack of robust CD31 immunoreactivity in cellular regions of xenografts (second column). Cleaved caspase 3 staining in proximal acellular regions (third column). Identification of regions of stress in vivo via detection of Pimonidazole adducts with Hypoxyprobe antibodies at the junction between cellular and acellular zones. All images are representative of multiple tumors assayed for each genotype. No notable differences were seen between ALK4^L75A^-Fc expressing tumors and controls for these characteristics. **Supplemental Fig. 4.** Altered signaling in ALK4^L75A^-Fc expressing xenografts**.** A diminution of phospho-AKT signaling can be discerned in ALK4^L75A^-Fc expressing tumors relative to mock tumors in both Hypoxyprobe positive and negative cellular regions (top row). Hypoxic regions in Mock tumors had generally diminished SMAD2/3 phosphorylation whereas ALK4^L75A^-Fc tumors often exhibited SMAD2/3 phosphorylation in hypoxic zones especially as these abut the acellular zones. All images are representative of three tumors assayed for each genotype. Scale bar= 50μm.

## Data Availability

All data generated or analyzed during this study are included in this published article, or available upon reasonable request from the corresponding author.
