# Supplementary material for: CRIPTO antagonist ALK4L75A-Fc inhibits breast cancer cell plasticity and adaptation to stress
Source: Breast Cancer Res. 2020 Nov 13;22:125. doi: 10.1186/s13058-020-01361-z (PMC7664111; doi:10.1186/s13058-020-01361-z)
Supplement: Supplementary file 1 — Additional file 1: Supplemental Fig. 1. A) Schematic of a lentiviral expression vector (S14) with fluorescent luminescent markers and doxycycline-inducible ALK4L75A-Fc. (B,C) Western blot demonstrating doxycycline-dependent expression of FLAG-tagged ALK4L75A-Fc in S14-transduced MDA-MB-231 cells, B and secretion into conditioned media, C. (D) Amplification curves for TDGF1 (ie CRIPTO) from equivalent starting material in MDA-MB231 cells transduced with a Dox dependent shCRIPTO vector. Supplemental Fig. 2. Morphology of organoids seeded into secondary cultures following treatment with Dox to induce ALK4L75A-Fc expression during primary organoid out growth. Supplemental Fig. 3. A) a panoramic view of a hematoxylin/Eosin stained MDA-MB-468 tumor section by which position and morphology can be used to assign regional and structural information. P=periphery, v=presumptive vasculature, C=cellular region, S =stressed zone (see B), A = acellular zone. No gross morphological differences apart from average size were noted for ALK4L75A-Fc expressing tumors relative to mock controls. (B) High magnification images of presumptive vasculature in MDA-MB-468 xenografts containing obvious red blood cells (left panels). Lack of robust CD31 immunoreactivity in cellular regions of xenografts (second column). Cleaved caspase 3 staining in proximal acellular regions (third column). Identification of regions of stress in vivo via detection of Pimonidazole adducts with Hypoxyprobe antibodies at the junction between cellular and acellular zones. All images are representative of multiple tumors assayed for each genotype. No notable differences were seen between ALK4L75A-Fc expressing tumors and controls for these characteristics. Supplemental Fig. 4. Altered signaling in ALK4L75A-Fc expressing xenografts. A diminution of phospho-AKT signaling can be discerned in ALK4L75A-Fc expressing tumors relative to mock tumors in both Hypoxyprobe positive and negative cellular regions (top row). Hypoxic [file 13058_2020_1361_MOESM1_ESM.docx]

Supplemental Figure 1


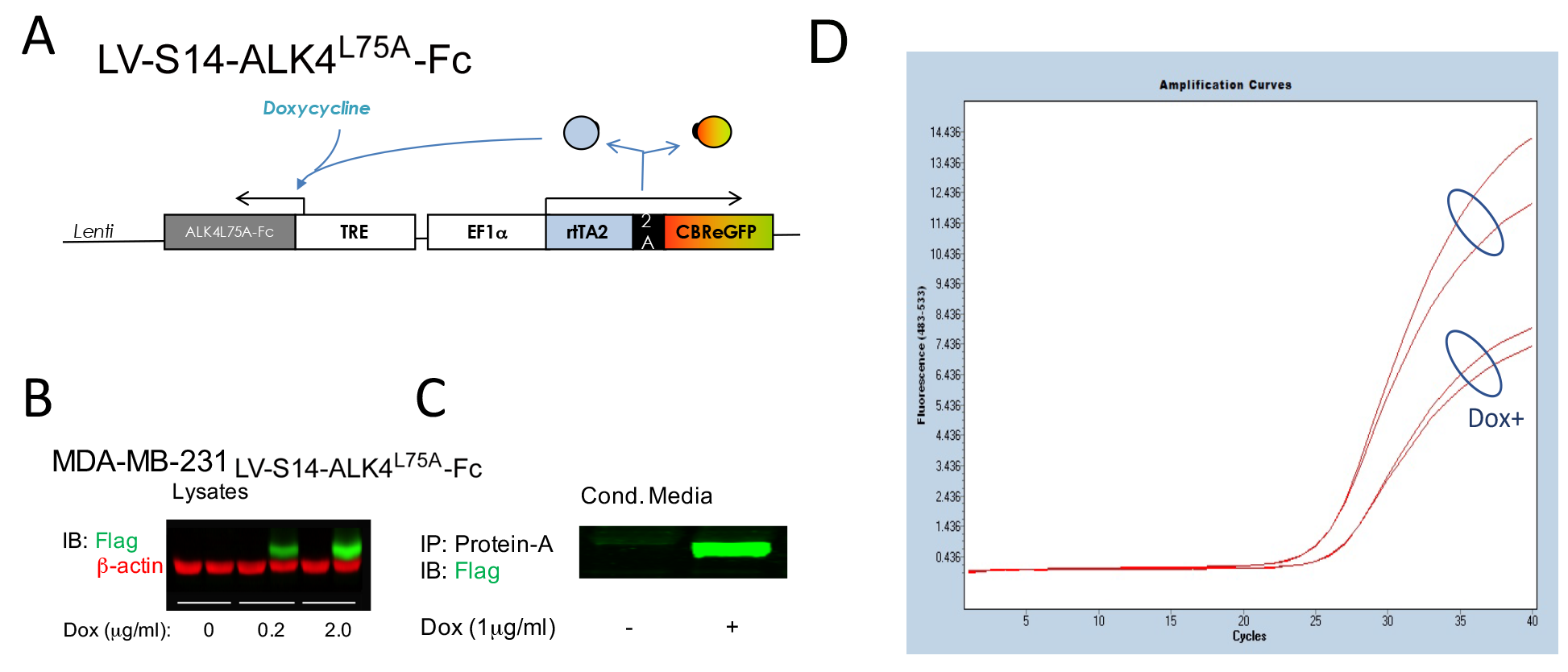


**Supplemental Fig. 1.** A) Schematic of a lentiviral expression vector (S14) with fluorescent luminescent markers and doxycycline-inducible ALK4^L75A^-Fc. (B,C) Western blot demonstrating doxycycline-dependent expression of FLAG-tagged ALK4^L75A^-Fc in S14-transduced MDA-MB-231 cells, B and secretion into conditioned media, C. (D) Amplification curves for TDGF1 (ie CRIPTO) from equivalent starting material in MDA-MB231 cells transduced with a Dox dependent shCRIPTO vector.

Supplemental Figure 2


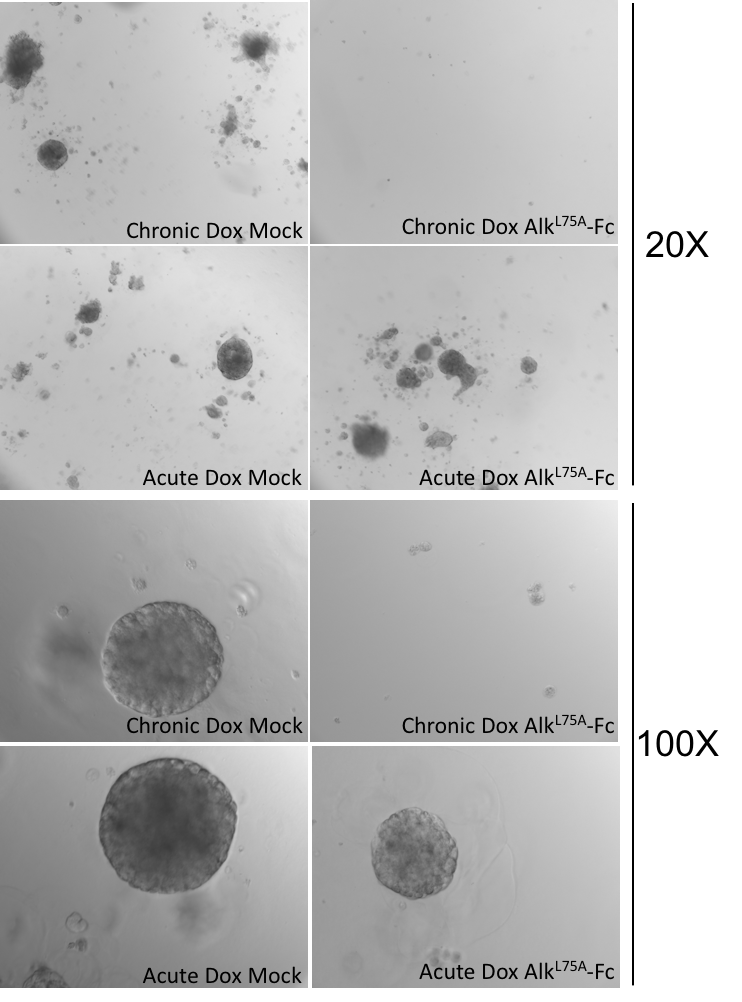


**Supplemental Fig. 2.** Morphology of organoids seeded into secondary cultures following treatment with Dox to induce ALK4^L75A^-Fc expression during primary organoid out growth.

Supplemental Figure 3


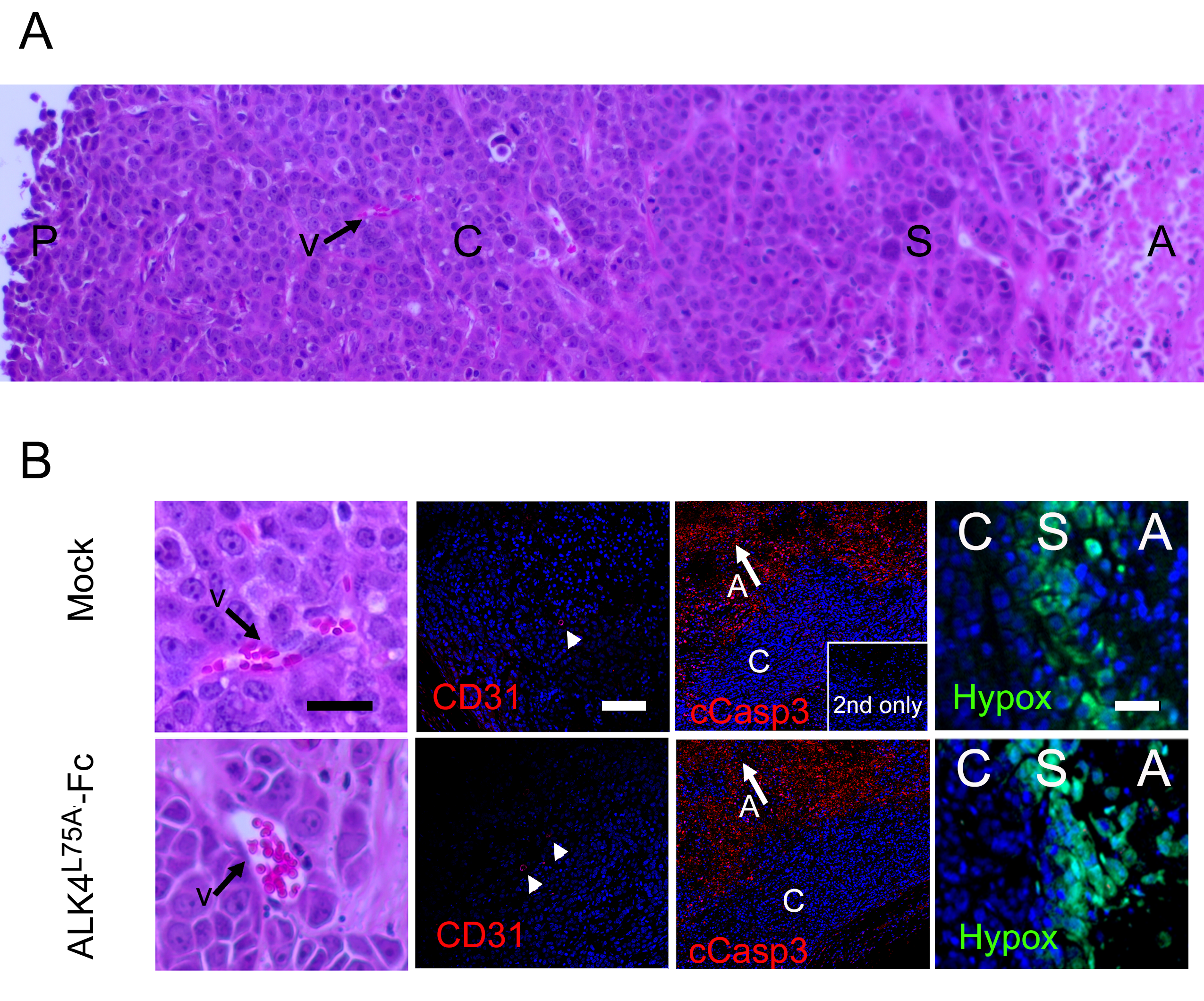


**Supplemental Fig. 3.** A) a panoramic view of a Hematoxylin/Eosin stained MDA-MB-468 tumor section by which position and morphology can be used to assign regional and structural information. P=periphery, v=presumptive vasculature, C=cellular region, S =stressed zone (see B), A = acellular zone. No gross morphological differences apart from average size were noted for ALK4L75A-Fc expressing tumors relative to mock controls. (B) High magnification images of presumptive vasculature in MDA-MB-468 xenografts containing obvious red blood cells (left panels). Lack of robust CD31 immunoreactivity in cellular regions of xenografts (second column). Cleaved caspase 3 staining in proximal acellular regions (third column). Identification of regions of stress in vivo via detection of Pimonidazole adducts with Hypoxyprobe antibodies at the junction between cellular and acellular zones. All images are representative of multiple tumors assayed for each genotype. No notable differences were seen between ALK4L75A-Fc expressing tumors and controls for these characteristics.

Supplemental Figure 4


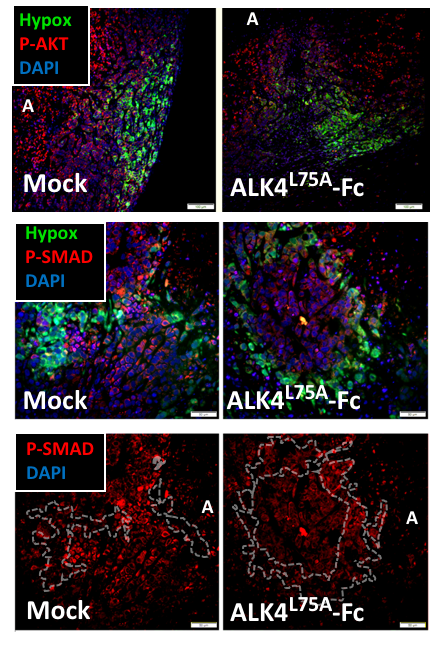


**Supplemental Fig. 4.** **Altered signaling in ALK4^L75A^-Fc expressing xenografts.** A diminution of phospho-AKT signaling can be discerned in ALK4^L75A^-Fc expressing tumors relative to mock tumors in both Hypoxyprobe positive and negative cellular regions (top row). Hypoxic regions in Mock tumors had generally diminished SMAD2/3 phosphorylation whereas ALK4^L75A^-Fc tumors often exhibited SMAD2/3 phosphorylation in hypoxic zones especially as these abut the acellular zones. All images are representative of three tumors assayed for each genotype. Scale bar= 50
